# ﻿Diversity, pathogenicity and two new species of pestalotioid fungi (Amphisphaeriales) associated with Chinese Yew in Guangxi, China

**DOI:** 10.3897/mycokeys.102.113696

**Published:** 2024-02-27

**Authors:** Yifeng Wang, Kin-Ming Tsui, Shimei Chen, Chongjuan You

**Affiliations:** 1 Beijing Key Laboratory for Forest Pest Control, Beijing Forestry University, Beijing 100083, China Beijing Forestry University Beijing China; 2 National Centre for Infectious Diseases, Tan Tock Seng Hospital, Singapore 308433, Singapore National Centre for Infectious Diseases, Tan Tock Seng Hospital Singapore Singapore; 3 Faculty of Medicine, University of British Columbia, Vancouver, V6T 1Z3, Canada University of British Columbia Vancouver Canada

**Keywords:** *Neopestalotiopsis*, *Pestalotiopsis*, phylogeny, taxonomy, two new species

## Abstract

Chinese yew, Taxuschinensisvar.mairei is an endangered shrub native to south-eastern China and is widely known for its medicinal value. The increased cultivation of Chinese yew has increased the incidence of various fungal diseases. In this study, Pestalotioid fungi associated with needle spot of Chinese yew were isolated from Guangxi Province. Based on morphological examinations and multi-locus (ITS, *tub2*, *tef-1α*) phylogenies, these isolates were identified to five species, including two new species, *Pestalotiopsistaxicola* and *P.multicolor*, two potential novel *Neopestalotiopsis* species, *Neopestalotiopsis* sp. 3 and *Neopestalotiopsis* sp. 4, with a known *Pestalotiopsis* species (*Pestalotiopsistrachycarpicola*), firstly recorded from Chinese yew. These two new *Pestalotiopsis* species were morphologically and phylogenetically distinct from the extant Pestalotioid species in Chinese yew. Pathogenicity and culture characteristic tests of these five Pestalotioid species were also performed in this study. The pathogenicity test results revealed that *Neopestalotiopsis* sp. 3 can cause diseases in Chinese yew needles. These results have indicated that the diversity of Pestalotioid species associated with Chinese yew was greater than previously determined and provided helpful information for Chinese yew disease diagnosis and management.

## ﻿Introduction

The Chinese yew (Taxuschinensisvar.mairei (Lemée et Lévl) Cheng et L.K. Fu) is an evergreen tall tree unique to south-eastern China and is widely known as a significant variety of medicinal plants because it contains taxol (paclitaxel) which is a natural antitumour drug with unique physiological functions in the bark, branches and leaves (Pyo et al. 2004; Li et al. 2008; Wu et al. 2013). Chinese yew has been listed on the IUCN Red List as an endangered species and has been under first-class protection in China, as it grows slowly and has poor regeneration ability, making it unable to meet strong medical demands (Ru 2006; Liu et al. 2019). Many natural reserves and conservation parks have been established to protect the Chinese yew. It has been widely cultivated in 17 provinces of China over the past few decades (Gao 2006; Zhang et al. 2018; Li et al. 2021a).

The expansion of Chinese yew cultivation in recent years has led to the emergence of various diseases that pose a significant threat to the Chinese yew industry (Zhang et al. 2018). Many fungal pathogens have been reported to cause Chinese yew diseases during their growth, such as needle spot caused by *Neopestalotiopsisclavispora* and *Pestalotiopsisaffinis* (Chen et al. 2002; Wang et al. 2019), needle blight caused by *Pestalotiopsismicrospora* (Li 2017), anthracnose disease caused by *Colletotrichumgloeosporioides* (Fan et al. 2006) and wilt disease caused by *Phoma* sp., while *Fusariumoxysporum* is responsible for root rot (Qian et al. 2015). Needle diseases caused by Pestalotioid fungal species (e.g. *N.clavispora*, *P.affinis* and *P.microspora*) are a serious threat to Chinese yew in Fujian and Yunnan Provinces, China (Chen et al. 2002; Li 2017; Wang et al. 2019). Up to 40% of the Chinese yew are severely affected in the field (Wang et al. 2019). The needles of Chinese yew became brown and reddish-brown from the leaf margin to the main vein, with irregular or round spots. Eventually, the spots coalesced and the needles withered and abscised (Li 2017; Wang et al. 2019). Jeon and Cheon (2014) reported that *P.microspora* also caused leaf blight in Japanese yew (*Taxuscuspidata* Sieb. & Zucc.) in South Korea. This disease developed on needles, particularly under high humidity, producing brown or tan spots that coalesce to form large lesions (Jeon and Cheon 2014).

Pestalotioid fungi (*Pestalotiopsis*-like fungi) represent an important fungal group that commonly occur as plant pathogens, endophytes and saprophytes in a wide range of hosts (Maharachchikumbura et al. 2014; Reddy et al. 2016; Ran et al. 2017; Freitas et al. 2019; Yang et al. 2021; Xiong et al. 2022). This group, which comprise *Neopestalotiopsis*, *Pestalotiopsis* and *Pseudopestalotiopsis*, belongs to the order Amphisphaeriales (Jiang et al. 2022; Zhang et al. 2022). The two genera *Neopestalotiopsis* and *Pseudopestalotiopsis* were segregated from *Pestalotiopsis* by Maharachchikumbura et al. (2014) based on multigene phylogenetic analysis and morphological differences (conidiogenous cells and colour intensities of the median conidial cell). *Pestalotiopsis* is characterised by lightly pigmented concolorous median cells, whereas *Neopestalotiopsis* species are characterised by versicolorous median cells and indistinct conidiophores and *Pseudopestalotiopsis* by darkly coloured concolorous median conidial cells (Maharachchikumbura et al. 2014). Many *Pestalotiopsis* species have been isolated from *Taxus* spp. as endophytes rich in secondary metabolites. For example, *Pestalotiopsisversicolor* has been isolated from healthy leaves of *Taxuscuspidata* and is an excellent alternative source of paclitaxel supply (Kumaran et al. 2010). Furthermore, *P.microspora*, which causes Chinese yew leaf blight, is an endophyte of the Himalayan yew (*Taxuswallichiana*), which produces taxol (Strobel et al. 1996).

During a survey of diseases in Chinese yew, moderate-to-severe incidences of needle spot and stem canker diseases were observed in some planting areas in Guangxi Province in 2020, and several Pestalotioid fungi were isolated from the diseased Chinese yew. The objectives of our study were to: (i) identify the Pestalotioid fungi recovered from symptomatic Chinese yew using morphological features and molecular data analyses; (ii) evaluate the pathogenicity of different fungal species on detached Chinese yew; and (iii) evaluate the effects of temperature, light duration and carbon source on the mycelial growth rate of different fungal species.

## ﻿Materials and methods

### ﻿Sample collections and Fungal Isolation

In May 2020, twenty symptomatic Chinese yew specimens were collected from the Guangxi Province. Small sections (5 × 5 mm) were cut from the margins of the infected needles and stems, surface-sterilised in 75% ethanol for 30 s, sterilised in 3% (vol/vol) sodium hypochlorite for 1 min, followed by three rinses in sterile distilled water and finally dried on sterilised filter paper (Rubini et al. 2005; Li et al. 2021b). The sections were plated on PDA plates and incubated at 25 °C. The isolates were aseptically transferred to fresh PDA and purified using a single-spore culture. The holotype specimens in this study are deposited in the Museum of Beijing Forestry University (Herbarium Code: BJFC). The pure fungal cultures in this study are deposited in China Forestry Culture Collection Center (Acronym: CFCC).

### ﻿Morphological and culture characterisation

The colony characteristics (colour and texture) of each isolate on PDA were observed after 7 d incubation at 25 °C and the morphological characters of conidiophores, conidiogenous cells and conidia of each isolate on PDA were observed after 14 d incubation at 25 °C (Li et al. 2021b). The size was determined by measuring the length and width of 50 randomly chosen conidia using an Olympus SZX2-FOF Light microscope (Tokyo, Japan) according to the method described by Shu et al. (2020). Descriptions, nomenclature and illustrations of taxonomic novelties have been deposited in MycoBank (https://www.mycobank.org/).

### ﻿DNA extraction and PCR amplification

Genomic DNA was extracted from 7 day-old colonies grown on PDA using the cetyltrimethylammonium bromide (CTAB) method (Doyle and Doyle 1990). Three loci were amplified: the internal transcribed spacer region of ribosomal DNA (ITS) with primers ITS1/ITS4 (White et al. 1990); beta-tubulin (*tub2*) with primers Bt2a/Bt2b (Glass and Donaldson 1995) and the translation elongation factor 1-alpha gene (*tef-1α*) with EF1-688F/EF1-1251R (Alves et al. 2008). The PCR mixture consisted of 10 μl TopTaq Master Mix, 7 μl nuclease-free H_2_O, 1 μl of each primer and 1 μl DNA samples were made up to the final volume of 20 μl. Primer sequences and reaction conditions were shown in Suppl. material 1. The PCR products were sent for sequencing at Beijing Tsingke Biotech Company, Ltd., Beijing, China. The DNA sequences have been deposited in GenBank (Suppl. material 2).

### ﻿Sequence alignment and phylogenetic analyses

The taxa used in the analyses were obtained from the sequence data of *Pestalotiopsis* and *Neopestalotiopsis* downloaded from GenBank (Suppl. material 2). Sequence alignments were performed using MAFFT v.7 (mafft.cbrc.jp/alignment/server) (Katoh and Standley 2013). Phylogenetic analyses, based on a combined dataset of ITS, *tub*2 and *tef-1α* sequence data, were performed with Maximum Parsimony (MP), Maximum Likelihood (ML) and Bayesian Inference (BI). MP analysis was performed with PAUP 4.0b using a heuristic search algorithm (1,000 random-addition sequences) with tree bisection and reconnection (TBR) branch swapping. MaxTrees were set to 5,000, branches of zero length were collapsed and all equally parsimonious trees were saved (Swofford 2003). ML analysis was performed using RAxML-HPC BlackBox v. 8.2.12, with 1000 rapid bootstrap replicate runs using the GTRGAMMA model of nucleotide evolution (Stamatakis 2014). For BI analysis, the best-fit evolutionary models for each partitioned locus were deduced on the AIC (ITS: GTR+I+G, *tub2*: HKY+I+G and *tef-1α*: TrN+I+G) which were estimated in MrModelTest v. 2.3. The BI analysis was performed using MrBayes v.3.1.2, with a Markov Chain Monte Carlo (MCMC) algorithm running from random trees for 1,000,000 generations. The resulting trees were edited using FigTree v.1.4.2 (Rambaut 2016) and Adobe Illustrator CS5.

### ﻿Culture characteristics

Five Pestalotioid species were selected to evaluate the effects of temperature, light duration and carbon source on mycelial growth. Mycelial plugs of each species were taken from the colony margins of 3-day-old cultures and transferred to fresh plates (20 ml of culture medium, pH 6). The effects of temperature were tested in the range 19 to 31 °C with a 3 °C gradient on PDA and incubated in dark. To monitor the effects of light duration, the isolates were tested under three diel light cycles (24 h dark, 12 h light/12 h dark and 24 h light per day) on PDA and incubated at 25 °C. The effects of the carbon source were tested using sucrose, maltose and dextrose with the same carbon content. Three replicates were used for the temperature and light duration tests and two replicates were used for the carbon test. The colony diameters were measured daily.

### ﻿Pathogenicity testing

Healthy needles were collected from five-year old and healthy Chinese yew grown in a greenhouse. The needles were washed with tap water, submerged in 70% ethanol for 2 min and rinsed twice with sterile water (Li et al. 2021b). Clean dry needles were placed on Petri dishes with moist filter paper underneath. Puncture wounds were made in the middle of each needle using a sterilised needle. The wounded needles were inoculated with mycelial PDA plugs (3 mm in diameter) and spore suspensions (18 µl, 10^6^ conidia/ml) of five Pestalotioid isolates, respectively. Three replicates were used for the mycelial PDA plug test and 10 replicates were used for the spore suspension test. Lesion length was recorded daily.

## ﻿Results

### ﻿Field observations and fungal isolation

**Figure 1.**
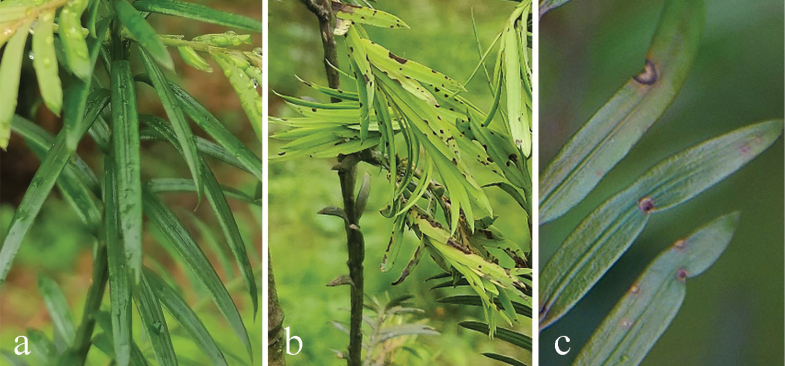
**a** healthy Chinese yew needles **b, c** chinese yew needles with spots.

A field survey was conducted from April to October 2020 in a forest farm in northern Guangxi, China, where more than 500 Chinese yew trees were maintained. Needle spot disease in Chinese yew causes serious damage, with an incidence of 58– 70% in plants. The spots on the needles were initially small, brown to black and oval to irregular. Subsequently, they gradually expand and finally coalesce, forming large black spots. In severe cases, lesions can develop in large portions of a single needle. Over time, the heavily infected leaves dried and died. Lesions were also visible on the stems and the spots on the stems darkened and became necrotic (Fig. 1).

Thirty-six isolates were obtained from the diseased needles and stems of Chinese yew and 18 isolates of Pestalotioid fungi were identified based on their culture characteristics and conidial morphology. Eight isolates were grouped into *Neopestalotiopsis* species with versicolorous median cells and ten isolates were identified as *Pestalotiopsis* with lightly pigmented concolorous median cells. Colonies of most Pestalotioid stains were initially whitish and later greyish or yellow on PDA. Conidia varied from 14.5 to 25.0 µm mean length and 3.5 to 8.0 µm mean width. The apical appendages showed the largest variation in size, with a mean length of 5.0 to 23.0 µm. The number of apical appendages varied between two and four, with three being the most common. Basal appendages were hyaline, straight or slightly curved and varied from 1.0 to 5.0 µm mean length.

### ﻿Phylogenetic analyses

**Figure 2.**
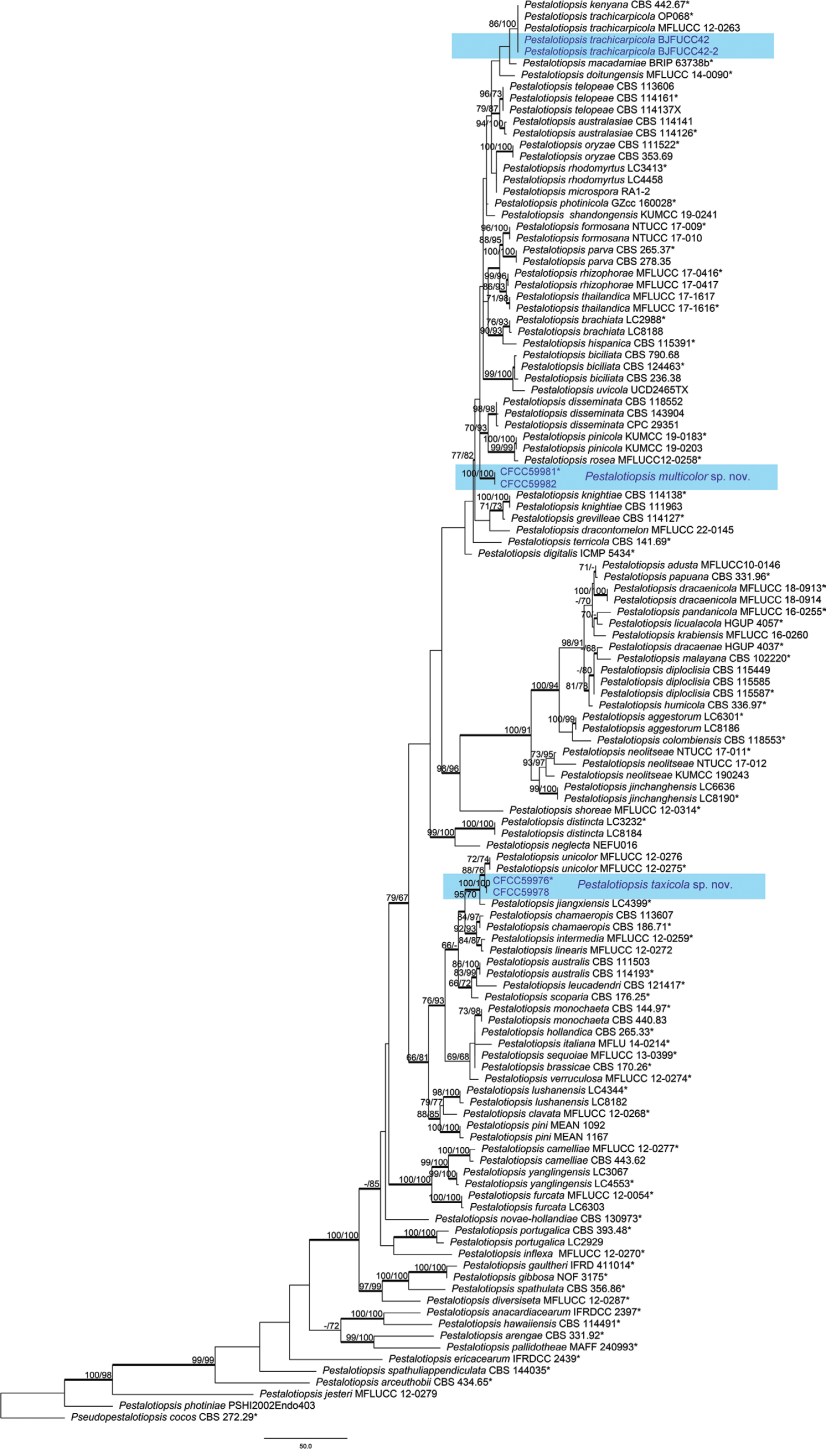
A phylogenetic tree of *Pestalotiopsis* generated from MP analysis, based on combined ITS, *tub*2 and *tef-1α* sequence data. Maximum Parsimony and Maximum Likelihood bootstrap values ≥ 65% are given at the nodes. The branches with Bayesian Inference posterior probabilities ≥ 0.90 are bold. Strains from this study are marked in blue. Ex-type strains are labelled with *.

The phylogenetic tree (*Pestalotiopsis*), based on the concatenated sequences of ITS, *tub2* and *tef-1α*, comprised 121 ingroups and one outgroup, *Pseudopestalotiopsiscocos* (CBS 272.29). A total of 1,496 characters including gaps (401 for ITS, 313 for *tub2* and 860 for *tef-1α*) were included in the phylogenetic analysis. Similar tree topologies were obtained using the MP, ML and BI methods and the best-scoring MP tree obtained from a heuristic search with 1000 random taxon additions is presented (Fig. 2). The phylogenetic tree placed the six *Pestalotiopsis* isolates into three well-supported monophyletic clades, representing two novel and one known species. Two of our isolates (CFCC59981 and CFCC59982), described as *Pestalotiopsismulticolor*, formed monophyletic groups with high support values (100% MP and ML, 1.00 BI). Two isolates (CFCC59976 and CFCC59978), identified as *Pestalotiopsistaxicola*, constituted an independent and strongly-supported subclade (100% MP and ML, 1.00 BI), sharing close affinity with *Pestalotiopsisunicolor* and *Pestalotiopsisjiangxiensis*. Two strains (BJFUCC42 and BJFUCC42-2) clustered with *Pestalotiopsistrachycarpicola* and were, therefore, described as known species.

**Figure 3.**
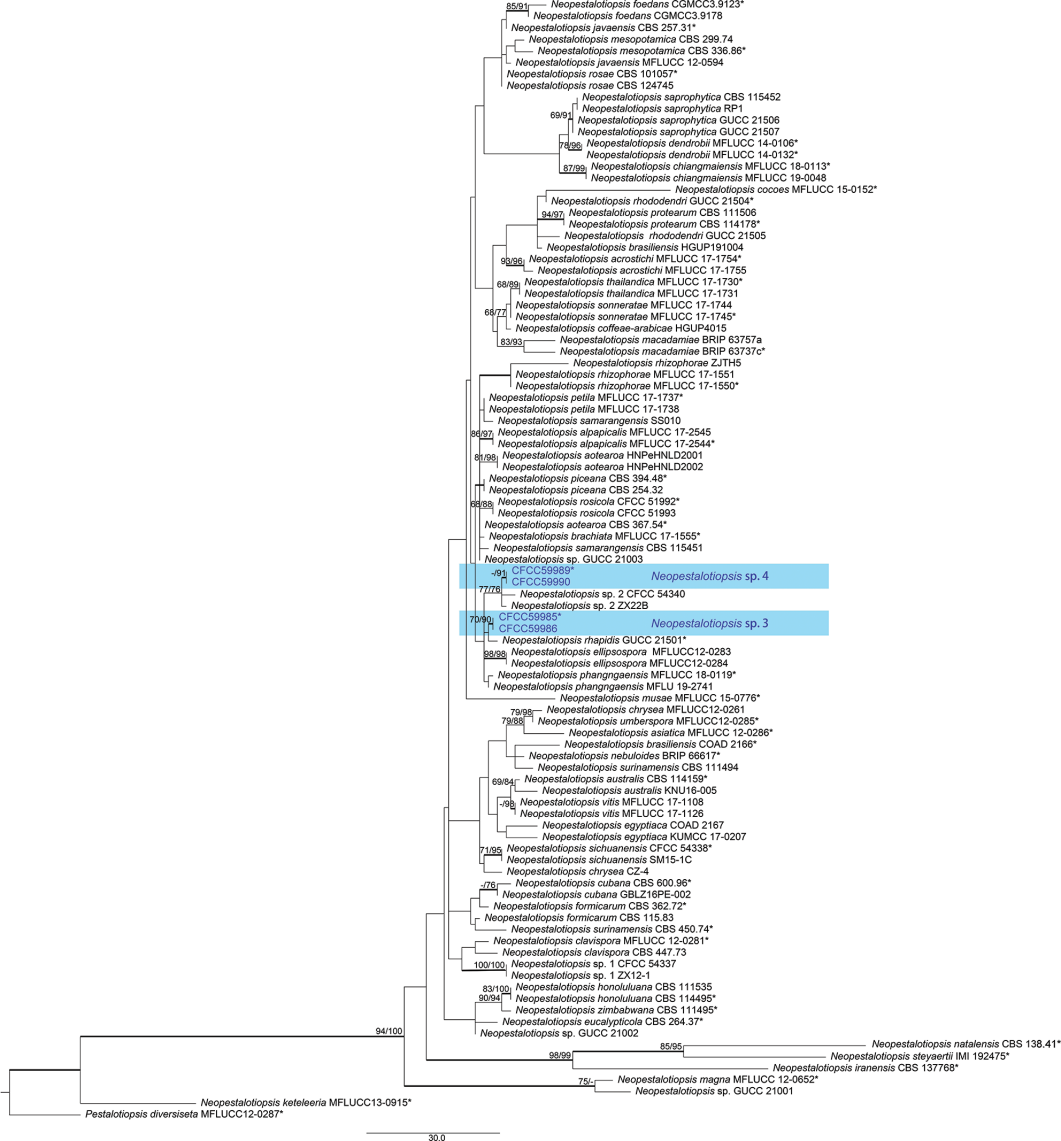
A phylogenetic tree of *Neopestalotiopsis* generated from MP analysis, based on combined ITS, *tub2* and *tef-1α* sequence data. Maximum Parsimony and Maximum Likelihood bootstrap values ≥ 65% are given at the nodes. The branches with Bayesian Inference posterior probabilities ≥ 0.90 are bold. Strains from this study are marked in blue. Ex-type strains are labelled with *.

The phylogenetic tree (*Neopestalotiopsis*), based on the concatenated gene sequences of ITS, *tub2* and *tef-1α*, comprised 95 ingroups and one outgroup, *Pestalotiopsisdiversiseta* (MFLUCC 12-0287). A total of 1, 386 characters including gaps (411 for ITS, 393 for *tub2* and 582 for *tef-1α*) were included in the phylogenetic analysis. Similar tree topologies were obtained using the MP, ML and BI methods and the best-scoring MP tree obtained from a heuristic search with 1000 random taxon additions is presented (Fig. 3). Two of our isolates (CFCC59989 and CFCC59990) clustered together and nested in a clade containing two different *Neopestalotiopsis* sp. 2 isolates (CFCC 54340 and ZX22B). In contrast, two isolates (CFCC59985 and CFCC59986), grouped *Neopestalotiopsisrhapidis* (Fig. 3).

### ﻿Taxonomy

#### ﻿ Pestalotiopsis trachycarpicola

Taxon classificationFungiAmphisphaerialesPestalotiopsidaceae

Yan M. Zhang & K.D. Hyde, 2012

2D9F5655-D139-51B5-9AB8-D120608C92C7

Fig. 4

##### Conidiogenesis.

**Figure 4.**
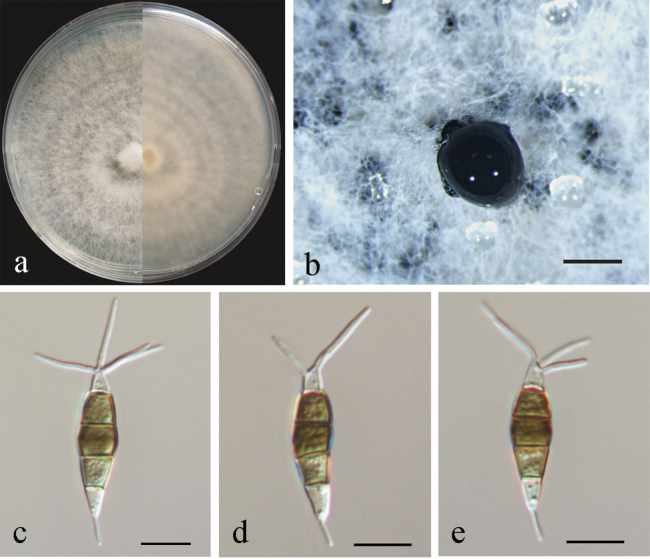
*Pestalotiopsistrachycarpicola* (BJFUCC42) **a** culture on PDA **b** conidiomata formed on PDA **c–e** conidia. Scale bars: 500 µm (**b**); 10 µm (**c–e**).

Conidiophores reduced to conidiogenous cells, indistinct. Conidiogenous cells were discrete, ampulliform, thin-walled, hyaline, smooth. Conidia fusiform to clavate, straight or slightly curved, olivaceous to brown, 4-septate, 18.5–25 × 4–6 μm, with apical and basal appendages. Basal cell obconic, hyaline, thin-walled, smooth, 3–5 µm; the three median cells dolioform, versicolor, pale brown to brown with septa darker than the rest of the cells, 11.5–13.5 µm, the second cell from base 3.5–6 µm; the third cell 3.5–4.5 µm; the fourth cell 3.5–5 µm; apical cell 2.5–4 µm, cylindrical, hyaline; 2–4 tubular apical appendages, arising from the apex of the apical cell each at different point, filiform, 5–15 µm; basal appendage present most of the time, single, tubular, unbranched, 3.5–4.5 µm (Fig. 4c-e). Sexual morph not observed.

##### Culture characteristics.

Colonies on PDA reaching 90 mm diameter after seven days at 25 °C, with an undulate and radial edge, with dense aerial mycelium on surface, white to faint yellow on front, pale honey-coloured on the reverse side (Fig. 4a). *Conidiomata acervular* in culture on PDA, globose, 100–500 μm in diameter, solitary or aggregated in clusters, exuding black conidial masses (Fig. 4b).

##### Material examined.

China, Guangxi Province, from diseased needles of Chinese yew, May 2020, Y. F. Wang (BJFC-S1955); living cultures BJFUCC42, BJFUCC42-2.

##### Notes.

*Pestalotiopsistrachycarpicola* was originally described from leaves of *Trachycarpusfortunei* in Kunming Botany Garden, Kunming, Yunnan Province, China (Zhang et al. 2012). In the present study, the two isolates clustered with *P.trachycarpicola* and *P.kenyana* with high support values (MP/ML = 86/100). *P.kenyana* (CBS 442.67, ex-type) and *P.trachicarpicola* (OP068, ex-type MFLUCC 12-0263) were demonstrated to be the same species, as there was no genetic distance between the two samples (Suppl. material 3). Morphologically, our new collections resembled *P.trachycarpicola* in colour and size of the conidiogenous cells, conidia and appendages (Zhang et al. 2012). Therefore, we reported the two isolates as a new host record of *P.trachycarpicola* from yews.

#### ﻿ Pestalotiopsis taxicola

Taxon classificationFungiAmphisphaerialesPestalotiopsidaceae

Y. F. Wang & C. J. You sp. nov.

FA6F18CB-9C97-57B9-B51C-F89F18A21173

MycoBank No: 847791

Fig. 5

##### Etymology.

Named after the host species, *Taxuschinensis*.

##### Holotype.

BJFC-S1954.

##### Conidiogenesis.

**Figure 5.**
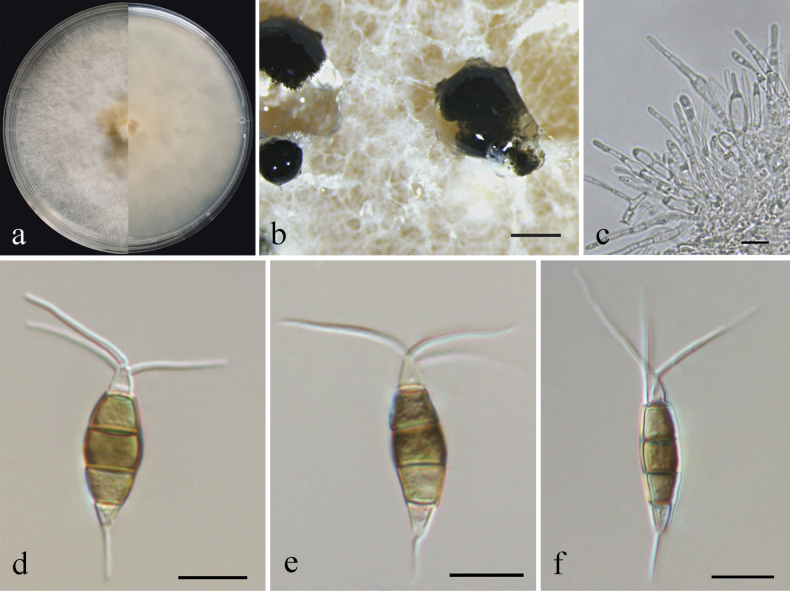
*Pestalotiopsistaxicola* (CFCC59976) **a** culture on PDA **b** conidiomata formed on PDA **c** conidiogenous cells **d–f** conidia. Scale bars: 500 µm (**b**); 10 µm (**c–f**).

Conidiophores reduced to conidiogenous cells, indistinct. Conidiogenous cells were discrete, ampulliform, thin-walled, hyaline, smooth. Conidia fusiform to clavate, straight or slightly curved, olivaceous to brown, 4-septate, 16.5–21 × 4–6 μm, with apical and basal appendages. Basal cell obconic, hyaline, thin-walled, smooth, 2.5–4 µm; the three median cells dolioform, versicolor, pale brown to brown with septa darker than the rest of the cells, 10.5–12 µm, the second cell from base 3–4 µm; the third cell 3.5–4 µm; the fourth cell 3.5–4 µm; apical cell 2.5–4 µm, cylindrical, hyaline; 3 tubular apical appendages, arising from the apex of the apical cell each at a different point, filiform, 9.5–15 µm; basal appendage present most of the time, single, tubular, unbranched, 2–5 µm (Fig. 5c-e). Sexual morph not observed.

##### Culture characteristics.

Colonies on PDA reaching 90 mm diameter after seven days at 25 °C, with an undulate and radial edge, with dense aerial mycelium on surface, initially yellow in the centre, becoming white at the margin, with white appressed mycelia radiating outwards (Fig. 5a). *Conidiomata acervular* in culture on PDA, globose, 200–700 μm in diameter, solitary or aggregated in clusters, exuding black conidial masses (Fig. 5b).

##### Material examined.

China, Guangxi Province, from diseased needles of Chinese yew, May 2020, Y. F. Wang (BJFC-S1954, holotype); ex-type living culture CFCC59976, living cultures CFCC59978, CFCC59979 and CFCC59980.

##### Notes.

*Pestalotiopsistaxicola* was phylogenetically placed in a clade encompassing *P.unicolor*, but as a unique lineage with high support (MP/ML = 100/100) (Fig. 2). Compared with *P.unicolor* (MFLUCC 12-0276, ex-type) and *P.taxicola* (CFCC59976, ex-type), there were one nucleotide difference in the ITS region and nine nucleotide differences in the *tef-1α* region. Morphologically, *P.taxicola* had smaller conidia (16.5–21 × 4–6 µm) than *P.unicolor* (20–24.5 × 4–6 µm). In addition, *P.taxicola* had only one basal appendage, whereas *P.unicolor* had 1–2 basal appendages (Maharachchikumbura et al. 2012). Therefore, the four isolates were designated as a new species.

#### ﻿ Pestalotiopsis multicolor

Taxon classificationFungiAmphisphaerialesPestalotiopsidaceae

Y. F. Wang & C. J. You sp. nov.

116300CA-148E-5169-885A-0736BC36B742

MycoBank No: 847792

Fig. 6

##### Etymology.

In reference to the multicoloured median cells.

##### Holotype.

BJFC-S1956.

##### Conidiogenesis.

**Figure 6.**
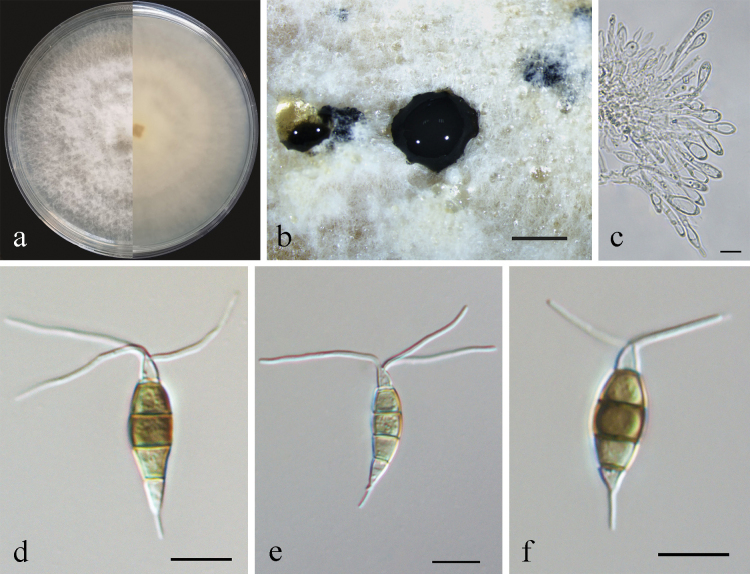
*Pestalotiopsismulticolor* (CFCC59981) **a** culture on PDA **b** conidiomata form on PDA **c** conidiogenous cells **d–f** conidia. Scale bars: 500 µm (**b**); 10 µm (**c–f**).

Conidiophores reduced to conidiogenous cells, short, subcylindrical and hyaline. Conidiogenous cells were discrete, ampulliform, thin-walled, hyaline, smooth. Conidia fusiform to clavate, straight or slightly curved, olivaceous to brown, 4-septate, 14.5–20 × 3.5–5.5 μm, with apical and basal appendages. Basal cell obconic, hyaline, thin-walled, smooth, 2–4.5 µm; the three median cells dolioform, versicolor, pale brown to brown with septa darker than the rest of the cells, 9.5–13 µm, the second cell from base 2.5–4.5 µm; the third cell 3–4.5 µm; the fourth cell 3–4 µm; apical cell 2.5–3.5 µm, cylindrical, hyaline; 2–3 (mostly 3) tubular apical appendages, arising from the apex of the apical cell each at a different point, filiform, 8–16 µm; basal appendage present most of the time, single, tubular, unbranched, 2.5–4.5 µm (Fig. 6c–e). Sexual morph not observed.

##### Culture characteristics.

Colonies on PDA reaching 90 mm diameter after seven days at 25 °C, with an undulate and radial edge, white aerial mycelium on surface flat or raised (Fig. 6a). *Conidiomata acervular* in culture on PDA, globose, 200–800 μm in diameter, solitary or aggregated in clusters, exuding black conidial masses (Fig. 5b).

##### Material examined.

China, Guangxi Province, from diseased needles of Chinese yew, May 2020, Y. F. Wang (BJFC-S1956, holotype); ex-type living culture CFCC59981, living cultures CFCC59982, CFCC59983, and CFCC59984.

##### Notes.

The two isolates (CFCC59981 and CFCC59982) formed a distinct lineage with high support values (MP/ML = 100/100) in the phylogenetic tree. The morphology of the two isolates was distinctive within the *Pestalotiopsis* genus because the conidia were far smaller than those of any other species. In addition, the colour of median cells in our new collections changed from light concolorous to versicolorous. Therefore, four isolates in the present study were designated as a new species.

#### ﻿ Neopestalotiopsis

Taxon classificationFungiAmphisphaerialesSporocadaceae

sp. 3

553E3A7B-7AF6-53BC-AB1F-578159E6A4B9

Fig. 7

##### Conidiogenesis.

**Figure 7.**
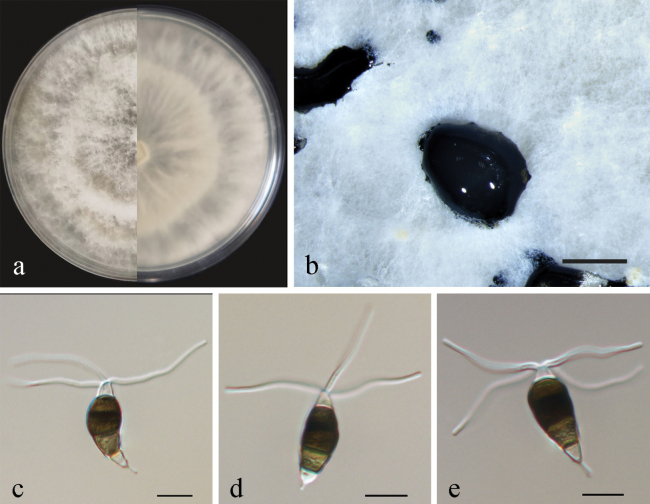
*Neopestalotiopsis* sp. 3 (CFCC59985) **a** culture on PDA **b** conidiomata form on PDA **c–e** conidia. Scale bars: 500 µm (**b**); 10 µm (**c–e**).

Conidiophores reduced to conidiogenous cells, hyaline, smooth. Conidiogenous cells were discrete, ampulliform, thin-walled, hyaline to light brown, smooth. Conidia fusiform to clavate, straight or slightly curved, olivaceous to brown, 4-septate, 15.5–19 × 6–7.5 μm, with apical and basal appendages. Basal cell obconic, hyaline, thin-walled, smooth, 2–4 µm; the three median cells 10.5–12 µm, dolioform, versicolor, pale brown to brown with septa darker than the rest of the cells, the second cell from base 2.5–4.5 µm; the third cell 3.5–4.5 µm; the fourth cell 3–4 µm; apical cell 1.5–3 µm, cylindrical, hyaline; 3–4 (mostly 3) tubular apical appendages, arising from the apex of the apical cell each at a different point, filiform, 16–23 µm; basal appendage present most of the time, single, tubular, unbranched, 1–2.5 µm (Fig. 7c–e). Sexual morph not observed.

##### Culture characteristics.

Colonies on PDA reaching 90 mm diameter after seven days at 25 °C, white aerial mycelium on surface, flat or raised, radiating outwards with an undulate and radial edge (Fig. 7a). *Conidiomata acervular* in culture on PDA, globose, 80–800 μm diameter, solitary or aggregated in clusters, exuding black conidial masses (Fig. 7b).

##### Material examined.

China, Guangxi Province, from diseased shoots of Chinese yew, May 2020, Y. F. Wang (BJFC-S1957); living cultures CFCC59985, CFCC59986, CFCC59987, CFCC59988.

##### Notes.

*Neopestalotiopsis* sp. 3 (CFCC59985 and CFCC59986) was phylogenetically close to *N.rhapidis* (Yang et al. 2021) (Fig. 3) but differed in conidial size (15.5–19 × 6–7.5 µm in *Neopestalotiopsis* sp. 3 vs. 22–25.5 × 4–6 µm in *N.rhapidis*). Furthermore, *Neopestalotiopsis* sp. 3 had longer apical appendages (16–23 µm vs. 11–16 µm) and shorter basal appendage (1–2.5 µm vs. 2–5.5 µm) than *N.rhapidis*. Compared with *N.rhapidis* (GUCC 21501, ex-type) and *Neopestalotiopsis* sp. 3, there were eleven nucleotide differences in the ITS region, four nucleotide differences in the *tef-1α* region and six nucleotide differences in the *tub2* region. In the genus *Neopestalotiopsis*, the interspecific relationship remains unclear (Li et al. 2021b), as some clades were not unanimously strongly supported, meaning the genus will be largely revised in the near future. Many taxa are likely to be synonymised in future publications. Therefore, CFCC59985 and CFCC59986 were not proposed as a new species.

#### ﻿ Neopestalotiopsis

Taxon classificationFungiAmphisphaerialesSporocadaceae

sp. 4

CEDB10C5-6EA7-5A6B-B6E0-339FC755F012

Fig. 8

##### Conidiogenesis.

**Figure 8.**
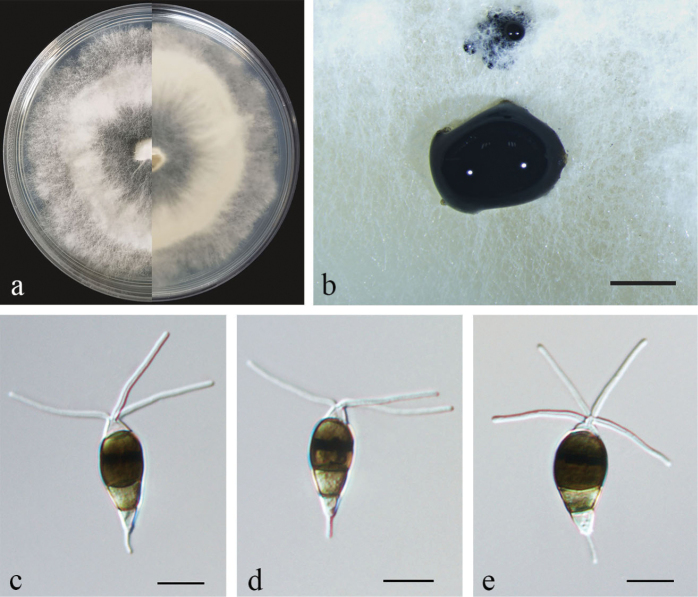
*Neopestalotiopsis* sp. 4 (CFCC59989) **a** culture on PDA **b** conidiomata form on PDA **c–e** conidia. Scale bars: 500 µm (**b**); 10 µm (**c–e**).

Conidiophores reduced to conidiogenous cells, hyaline, smooth. Conidiogenous cells were discrete, ampulliform, thin-walled, hyaline to light brown, smooth. Conidia fusiform to clavate, straight or slightly curved, olivaceous to brown, 4–septate, 15.5–18.5 × 6–8 μm, with apical and basal appendages. Basal cell obconic, hyaline, thin-walled, smooth, 2.5–4 µm; the three median cells 10.5–12 µm, dolioform, versicolor, pale brown to brown with septa darker than the rest of the cells, the second cell from base 3–3.5 µm; the third cell 3–4.5 µm; the fourth cell 3–5.5 µm; apical cell 1–2.5 µm, cylindrical, hyaline; 3–4 (mostly 3) tubular apical appendages, arising from the apex of the apical cell each at a different point, filiform, 9.5–15.5 µm; basal appendage present most of the time, single, tubular, unbranched, 1.5–4.5 µm (Fig. 8c–e). Sexual morph not observed.

##### Culture characteristics.

Colonies on PDA reaching 90 mm diameter after seven days at 25 °C, radiating outwards with an undulate and radial edge, white aerial mycelium was flat on the centres of the colony, while raised sharply in the outer ring (Fig. 8a). *Conidiomata acervular* in culture on PDA, globose, 100–900 μm diameter, solitary or aggregated in clusters, exuding black conidial masses (Fig. 8b).

##### Material examined.

China, Gungxi Province, from diseased branches of Chinese yew, May 2020, Y. F. Wang (BJFC-S1958); living cultures CFCC59989, CFCC59990, CFCC59991 and CFCC59992.

##### Notes.

*Neopestalotiopsis* sp. 4 (CFCC59989 and CFCC59990) was phylogenetically placed in a clade encompassing two *Neopestalotiopsis* sp. 2 isolates (CFCC54340 and ZX22B) from Yaan City, Sichuan Province (Jiang et al. 2021) (Fig. 3). Morphologically, *Neopestalotiopsis* sp. 4 had shorter conidia and longer apical appendages (15.5–18.5 × 6–8 µm, 9.5–15.5 µm) than CFCC54340 and ZX22B (22–25.2 × 6.2–7.7 µm, 5–10 µm). In addition, *Neopestalotiopsis* sp. 4 had more tubular apical appendages (3–4, mostly 3) than *Neopestalotiopsis* sp. 2 (2, seldom 3). Compared with *Neopestalotiopsis* sp. 2 (ZX22B) and *Neopestalotiopsis* sp. 4, there were six nucleotide differences in the ITS region, five nucleotide differences in the *tef-1α* region and three nucleotide differences in the *tub2* region. The taxonomy of the genera and species in this group remains unclear (Li et al. 2021b), as some clades were not unanimously strongly supported, meaning the genus will be largely revised in near future. Many taxa are likely to be synonymised in future publications. Therefore, CFCC59989 and CFCC59990 were not proposed as a new species.

### ﻿Culture characteristics

**Figure 9.**
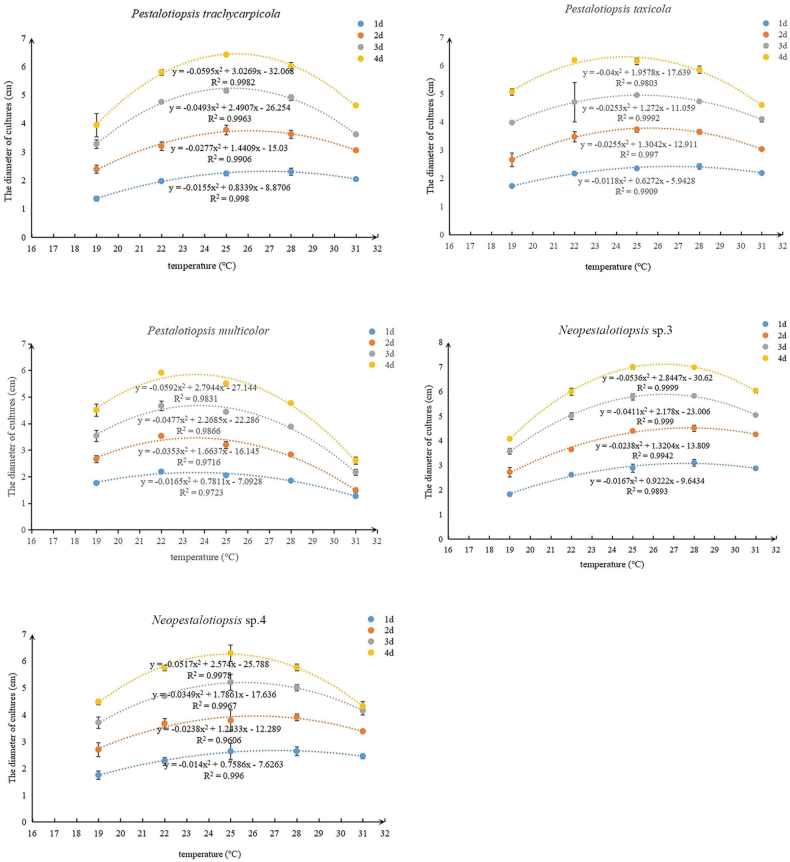
Effects of temperature on growth of isolates.

The effects of temperature on the growth of the five Pestalotioid species were shown in Fig. 9. When grown on PDA in the dark, all isolates grew at a temperature range of 19 to 31 °C, with optimum growth between 22 °C and 28 °C. The growth rate was drastically reduced below 22 °C and started to decline above 28 °C. The regression analysis showed the optimum temperatures for *P.trachycarpicola*, *P.taxicola*, *P.multicolor*, *Neopestalotiopsis* sp. 3 and *Neopestalotiopsis* sp. 4 were 25 °C, 24–26 °C, 22–25 °C, 25–28 °C and 25 °C, respectively. Within four days, the diameter of the majority of the isolates was approximately 90 mm, which is the diameter of the PDA plate.

Light duration had no effect on the mycelial growth of the two Pestalotioid species (*P.trachycarpicola* and *P.muticolor*) and the best growth of the three Pestalotioid fungi (*P.taxicola*, *P.multicolor* and *Neopestalotiopsis* sp. 4) was observed under continuous light (Suppl. material 4). The growth rate was highest for *P.taxicola* in 24 h light, which reached 66.4 mm diameter after four days. The growth rate was lowest for *P.trachycarpicola* in 12 h light/12 h dark, which reached only 55.5 mm in diameter after four days.

Five Pestalotioid species grew on all three tested carbon sources (Suppl. material 5). Maltose utilisation was the least efficient and the medium with dextrose showed comparatively high growth of the three tested fungi (*P.trachycarpicola*, *P.multicolor* and *Neopestalotiopsis* sp. 3). For *P.taxicola*, the utilisation of dextrose appeared to be higher than that of sucrose.

### ﻿Pathogenicity assay

**Figure 10.**
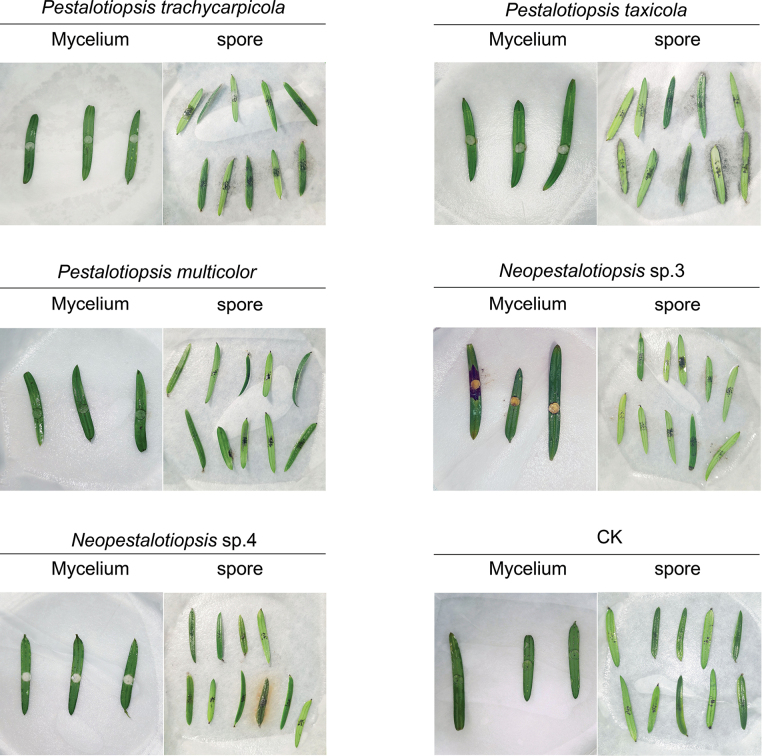
Lesions of the Chinese yew needles after 20 days. CK is the blank control. The black spots in the mid-region of the needles are puncture wounds made by sterilised needles.

The pathogenicity of two *Neopestalotiopsis* isolates (*Neopestalotiopsis* sp. 3 and *Neopestalotiopsis* sp. 4) and three *Pestalotiopsis* isolates (*P.trachycarpicola*, *P.taxicola* and *P.multicolor*) was tested by inoculating detached healthy needles according to Keith et al. (2003). Dark brown lesions and a necrotic zone, which resembled the symptoms that occurred in the field, were observed on the needles 20 days after inoculation with mycelium plugs of *Neopestalotiopsis* sp. 3, which did not occur in the control inoculated with agar media or with the other four Pestalotioid species (Fig. 10). *Neopestalotiopsis* sp. 3 was re-isolated from the infected needles and was confirmed to be the same as the inoculated pathogen through morphological and phylogenetic analyses. However, none of the five isolates produced typical symptoms in the wounded needles inoculated with the spore suspension (Fig. 10). The results revealed that only *Neopestalotiopsis* sp. 3 is pathogenic to Chinese yew needles.

## ﻿Discussion

Cultivation of Chinese yew in some regions (e.g. Guangxi) has resulted in an increase in yew diseases, including new needle spot diseases, which have caused great losses in production. However, very little is known about the pathogens and the presence and prevalence of needle diseases in Chinese yew (Wang et al. 2019). During the investigation of Pestalotioid fungi isolated from diseased Chinese yew in the Guangxi Province of China, *Neopestalotiopsis* sp. 3 was found to cause needle spots on Chinese yew. Two new species of *Pestalotiopsis* were identified in association with symptomatic Chinese yew, namely, *P.taxicola* and *P.multicolor* and one known species, *P.trachycarpicola*, was identified, based on morphology and multi-locus phylogeny. The results indicated that the diversity of Pestalotioid species associated with Chinese yew was greater than previously determined.

In the present study, the two species were distinct from other Pestalotioid species found in Chinese yew (Chen et al. 2002; Ding et al. 2009; Liu et al. 2010; Ismail et al. 2013; Li 2017; Subban et al. 2019; Qi et al. 2022; Sun et al. 2023). Morphologically, the conidia of *P.taxicola* and *P.multicolor* are smaller (16.5–21 × 4–6 μm and 14.5–20 × 3.5–5.5 μm, respectively). Phylogenetically, the two novel species in the present study were distantly related to four known Pestalotioid species, with the exception of *P.affinis*, which was known only from its morphological descriptions; there were no DNA-based sequence data to compare the phylogenetic relationship with our novel species (Chaiwan et al. 2020).

Pestalotioid fungi represent a diverse group of more than 593 taxa (http://www.indexfungorum.org/). However, the taxonomy of the genera and species in this group remains unclear (Maharachchikumbura et al. 2014). In recent years, multigene phylogenetic analyses (Combined ITS, *tub2* and *tef-1α* sequence data) have been increasingly utilised to distinguish taxa in the group (Maharachchikumbura et al. 2012). In this study, the low statistical support for some species in the phylogenetic tree of *Neopestalotiopsis* may be due to the absence of *tub2* and *tef-1α* data in some taxa (Yang et al. 2021). The interspecific relationships in the genus *Neopestalotiopsis* are ambiguous (Li et al. 2021b), as some clades are not unanimously strongly supported, indicating that the relationships amongst different species should be revised and re-evaluated. We decided against describing *Neopestalotiopsis* sp. 3 and sp. 4 as novel species in the genus. These two potentially novel species may be synonymised with existing species during revision and/or monography. Additional examination of archived specimens and sequence data is required for a better resolution and circumscription of *Neopestalotiopsis*.

In the present study, *P.trachycarpicola* was isolated from diseased needles. This is the first report of *P.trachycarpicola* isolated from yews in China. Under the trial conditions, no symptom development occurred in any of the inoculated needles, suggesting that *P.trachycarpicola* may behave as an endophyte in Chinese yew. *P.trachycarpicola* was first described in *Trachycarpusfortunei* in the Yunnan Province of China, causing leaf spot (Zhang et al. 2012) and has already been isolated from a wide range of hosts and locations worldwide (Fan et al. 2021; Khalilabad and Fotouhifar 2022; Araujo et al. 2023; Lan et al. 2023). For example, this fungus has been identified as the causal agent of leaf spots on *Sorghumbicolor* and *Panaxnotoginseng* in China (Fan et al. 2021; Lan et al. 2023), *Codiaeumvariegatum* in Iran (Khalilabad and Fotouhifar 2022) and blueberry in Brazil (Araujo et al. 2023).

In the present study, various *Neopestalotiopsis* and *Pestalotiopsis* species were isolated from infected Chinese yew samples with similar symptoms. Moreover, pathogenicity tests showed that only *Neopestalotiopsis* sp. 3 was pathogenic to Chinese yew, causing dark brown lesions on wounded needles, suggesting that *Neopestalotiopsis* sp. 3 is responsible for needle-spot disease in Chinese yew. The four other Pestalotioid fungi that do not produce typical disease symptoms may be due to their relatively low virulence or endophytes (Reddy et al. 2016; Ma et al. 2019). Alternatively, the development of needle spots disease is the result of more than one biotic or abiotic factor. Different studies have indicated that some *Neopestalotiopsis* and *Pestalotiopsis* species have been isolated from healthy and dead tissues and reported as endophytic fungi. Several *Pestalotiopsis* species have been isolated as endophytes, but have been reported as pathogens in various hosts and in different regions (Moslemi and Taylor 2015; Nozawa et al. 2019; Wang et al. 2019; Chen et al. 2021; Jiang et al. 2021). This suggests that some species may have interchangeable lifestyles such as endophytes, pathogens and saprobes within the same host. Understanding the external factors that influence fungal lifestyles can have major implications for plant health (Sun et al. 2023). Furthermore, the inoculation of wounded needles with mycelial plugs and spore suspensions of *Neopestalotiopsis* sp. 3 was found to be ineffective against disease infection and development. In future studies, the inoculum form and field conditions, such as relative humidity and temperature, should be considered. Furthermore, *Neopestalotiopsis* sp. 3 grew faster than other Pestalotioid species on all three carbon sources tested (p < 0.01), possibly contributing to its greater virulence than the others, but its growth on dextrose was not significantly different from that on the other two carbon sources (sucrose and maltose). In the present study, our results showed that temperature affected mycelial growth and *Neopestalotiopsis* sp. 3 grew well at 25–28 °C. The average monthly temperature between May and September is 25.6 °C and 26.8 °C, respectively, at our study site (http://www.weather.com.cn/). This is consistent with the most severe epidemic incidence and events observed during field surveillance and observation. Therefore, the culture characteristics of pathogenic fungi can be utilised to forecast and model disease spread.

In conclusion, the present study illustrates the diversity of Pestalotioid species associated with needle spot disease in Chinese yew. Understanding the taxonomy, biology and pathogenicity of Pestalotioid species associated with the Chinese yew will provide a foundation for monitoring disease development and provide information for management strategies for these pathogens.

## Supplementary Material

XML Treatment for Pestalotiopsis trachycarpicola

XML Treatment for Pestalotiopsis taxicola

XML Treatment for Pestalotiopsis multicolor

XML Treatment for Neopestalotiopsis

XML Treatment for Neopestalotiopsis

## ﻿Additional information

### Author ORCIDs

Yifeng Wang https://orcid.org/0009-0006-6533-6615

Kin-Ming Tsui https://orcid.org/0000-0001-5129-1037

Shimei Chen https://orcid.org/0009-0000-9862-9453

Chongjuan You https://orcid.org/0000-0001-6130-5703

### Data availability

All of the data that support the findings of this study are available in the main text or Supplementary Information.

## Supplementary materials

Supplementary material 1 PCR primer and PCR reaction conditions Data type xlsx Explanation note **table S1.** PCR primer and PCR reaction conditions. https://binary.pensoft.net/file/993287This dataset is made available under the Open Database License (http://opendatacommons.org/licenses/odbl/1.0/). The Open Database License (ODbL) is a license agreement intended to allow users to freely share, modify, and use this Dataset while maintaining this same freedom for others, provided that the original source and author(s) are credited.Yifeng Wang, Kin-Ming Tsui, Shimei Chen, Chongjuan You

Supplementary material 2 Specimen information and GenBank accession numbers Data type xlsx Explanation note **table S2.** Isolates and GenBank accession numbers of sequences used in this study (the isolates from this study are marked in red). https://binary.pensoft.net/file/993288This dataset is made available under the Open Database License (http://opendatacommons.org/licenses/odbl/1.0/). The Open Database License (ODbL) is a license agreement intended to allow users to freely share, modify, and use this Dataset while maintaining this same freedom for others, provided that the original source and author(s) are credited.Yifeng Wang, Kin-Ming Tsui, Shimei Chen, Chongjuan You

Supplementary material 3 Evolutionary divergence between sequences Data type xls Explanation note **table S3.** Estimates of Evolutionary Divergence between Sequences. https://binary.pensoft.net/file/993289This dataset is made available under the Open Database License (http://opendatacommons.org/licenses/odbl/1.0/). The Open Database License (ODbL) is a license agreement intended to allow users to freely share, modify, and use this Dataset while maintaining this same freedom for others, provided that the original source and author(s) are credited.Yifeng Wang, Kin-Ming Tsui, Shimei Chen, Chongjuan You

Supplementary material 4 Effects of light on growth of isolates Data type jpg Explanation note **fig. S1.** Effects of light on growth of isolates. https://binary.pensoft.net/file/993290This dataset is made available under the Open Database License (http://opendatacommons.org/licenses/odbl/1.0/). The Open Database License (ODbL) is a license agreement intended to allow users to freely share, modify, and use this Dataset while maintaining this same freedom for others, provided that the original source and author(s) are credited.Yifeng Wang, Kin-Ming Tsui, Shimei Chen, Chongjuan You

Supplementary material 5 Effects of carbon sources on growth of isolates Data type jpg Explanation note **fig. S2.** Effects of carbon sources on growth of isolates. https://binary.pensoft.net/file/993291This dataset is made available under the Open Database License (http://opendatacommons.org/licenses/odbl/1.0/). The Open Database License (ODbL) is a license agreement intended to allow users to freely share, modify, and use this Dataset while maintaining this same freedom for others, provided that the original source and author(s) are credited.Yifeng Wang, Kin-Ming Tsui, Shimei Chen, Chongjuan You

Supplementary material 6 Nucleotide differences between the isolates in this study Data type pdf Explanation note documentation S1: Nucleotide differences between the isolates in this study. https://binary.pensoft.net/file/993292This dataset is made available under the Open Database License (http://opendatacommons.org/licenses/odbl/1.0/). The Open Database License (ODbL) is a license agreement intended to allow users to freely share, modify, and use this Dataset while maintaining this same freedom for others, provided that the original source and author(s) are credited.Yifeng Wang, Kin-Ming Tsui, Shimei Chen, Chongjuan You

Supplementary material 7 Sequence data of the isolates in this study Data type zip Explanation note zip package S1: Sequence data of the isolates in this study. https://binary.pensoft.net/file/993293This dataset is made available under the Open Database License (http://opendatacommons.org/licenses/odbl/1.0/). The Open Database License (ODbL) is a license agreement intended to allow users to freely share, modify, and use this Dataset while maintaining this same freedom for others, provided that the original source and author(s) are credited.Yifeng Wang, Kin-Ming Tsui, Shimei Chen, Chongjuan You
