# Supplementary figures and images for: ﻿Diversity, pathogenicity and two new species of pestalotioid fungi (Amphisphaeriales) associated with Chinese Yew in Guangxi, China
Source: MycoKeys. 2024 Feb 27;102:201–24. doi: 10.3897/mycokeys.102.113696 (PMC10915749; doi:10.3897/mycokeys.102.113696)

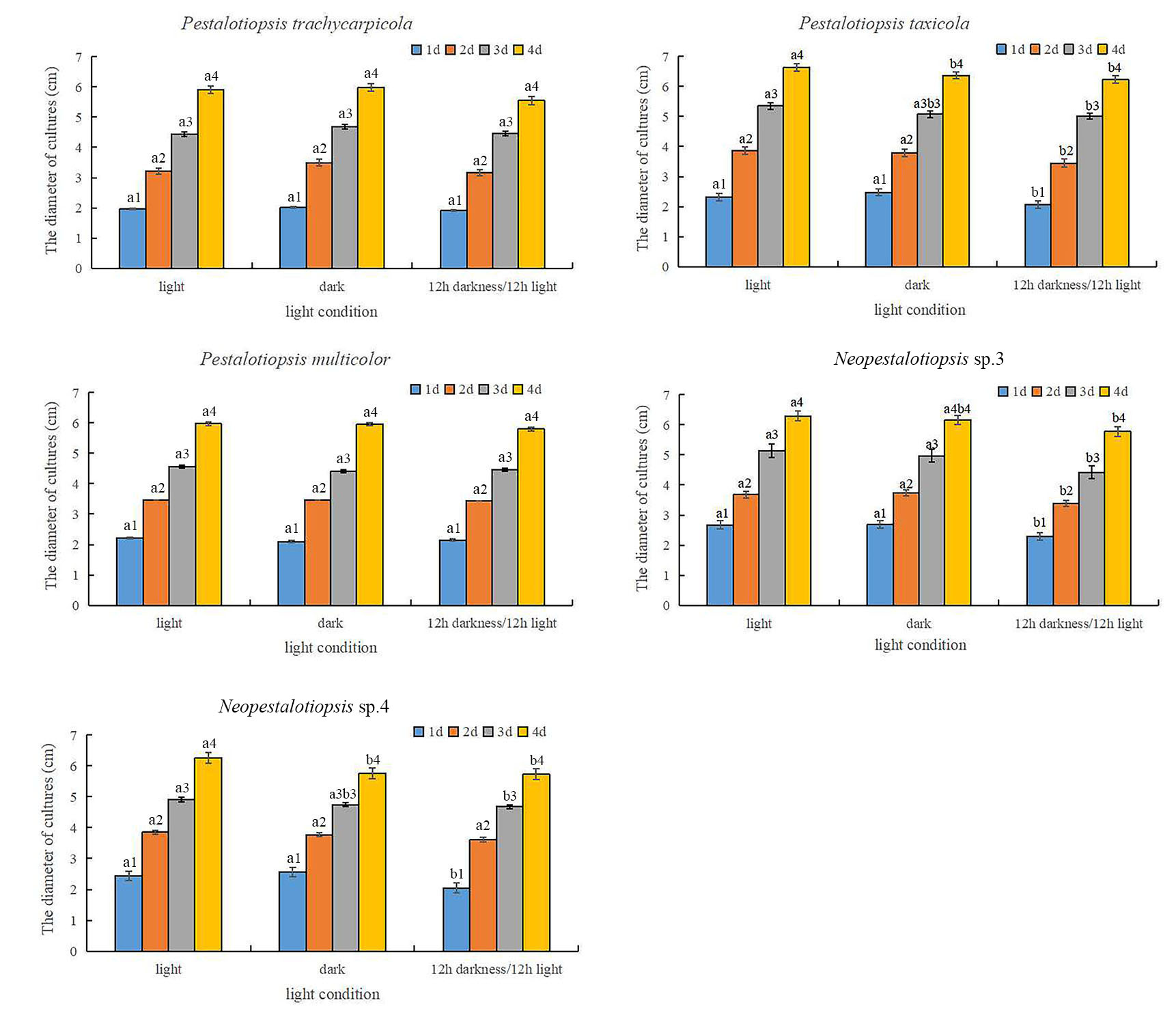

Supplement: Supplementary material 4 — Effects of light on growth of isolates [file mycokeys-102-201-s004.jpg]

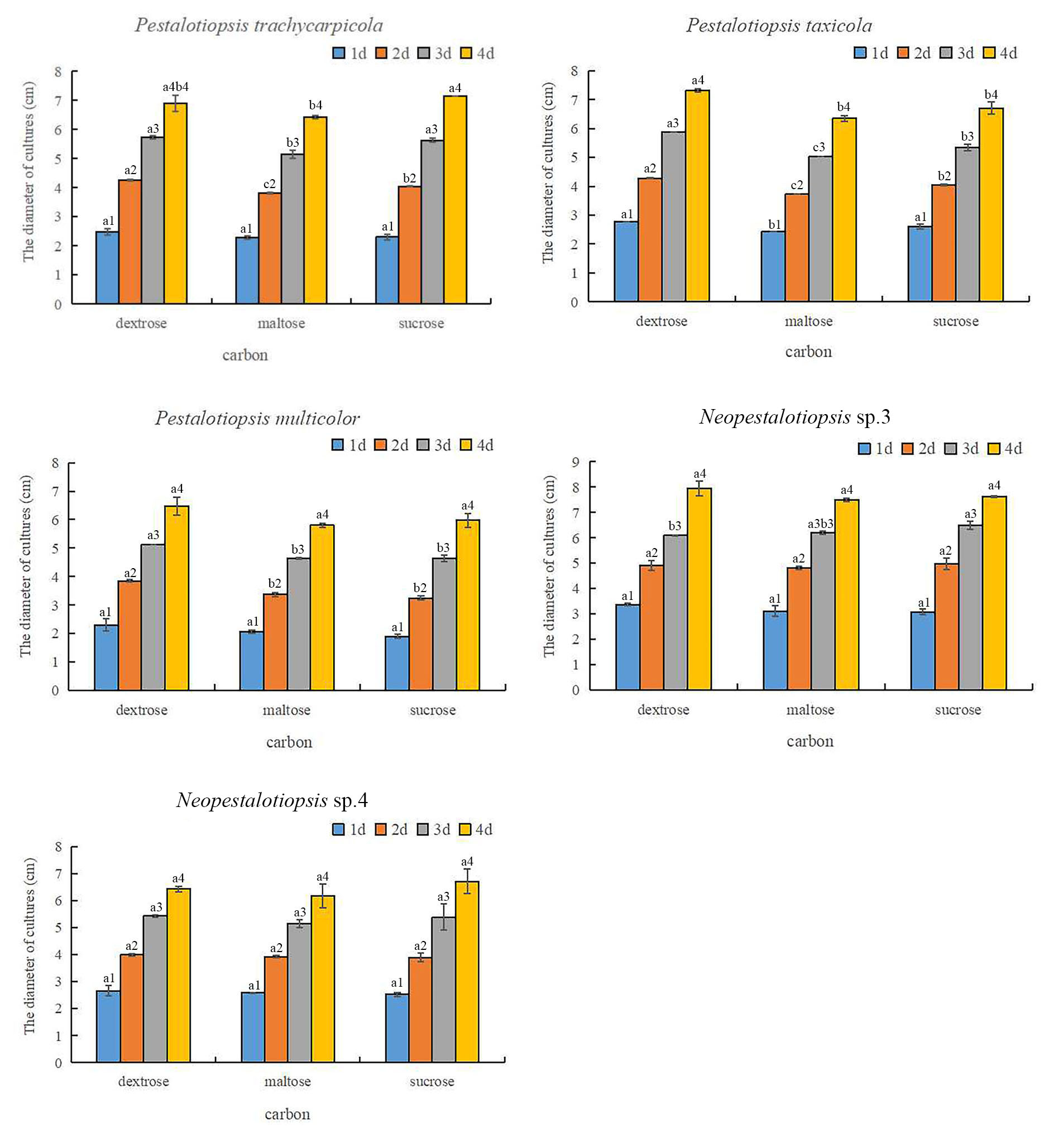

Supplement: Supplementary material 5 — Effects of carbon sources on growth of isolates [file mycokeys-102-201-s005.jpg]
