# Supplementary material for: ﻿Diversity, pathogenicity and two new species of pestalotioid fungi (Amphisphaeriales) associated with Chinese Yew in Guangxi, China
Source: MycoKeys. 2024 Feb 27;102:201–24. doi: 10.3897/mycokeys.102.113696 (PMC10915749; doi:10.3897/mycokeys.102.113696)
Supplement: Supplementary material 6 — Nucleotide differences between the isolates in this study [file mycokeys-102-201-s006.pdf]

Supplementary Material 6 Documentation S1: Nucleotide differences between the isolates in this study

We compared the nucleotide differences between the isolates in this study to confirm that they are not clones.

Between BJFUCC41 and BJFUCC41-2, there were 2 character differences in the ITS region (sites: 23, 590).

Between BJFUCC61 and BJFUCC61-2, there were 1 character difference in the ITS region (site: 606), 1 character difference in the *tub2* region (site: 442), and 1 character difference in the *tef-1α* region (site: 556).

Between BJFUCC52 and BJFUCC52-2, there was 1 character difference in the ITS region (site: 501), 1 character difference in the *tub2* region (site: 48).

Between BJFUCC83 and BJFUCC83-2, there were 1 character difference in the ITS region (site: 591), 2 character differences in the *tub2* region (sites: 443, 444), and 1 character difference in the *tef-1α* region (site:529).
